# Temperature rising would slow down tropical forest dynamic in the Guiana Shield

**DOI:** 10.1038/s41598-019-46597-8

**Published:** 2019-07-15

**Authors:** Mélaine Aubry-Kientz, Vivien Rossi, Guillaume Cornu, Fabien Wagner, Bruno Hérault

**Affiliations:** 1 Univ Guyane, UMR ‘Ecologie des Forêts de Guyane’ (AgroParisTech, Cirad, CNRS, Inra, Univ Antilles), Kourou, 97310 France; 2 AMAP, IRD, CNRS, CIRAD, INRA, Univ Montpellier, Montpellier, France; 3 Cirad, Univ Montpellier, UR Forests and Societies, Montpellier, France; 4 Université de Yaoundé I, UMMISCO (UMI 209), Yaoundé, BP337 Cameroon; 5 Remote sensing division, National Institute for space research-INPE, São José dos Campos, SP Brazil; 6 Institut National Polytechnique Félix Houphouët-Boigny (INP-HB), Yamoussoukro, Côte d’Ivoire

**Keywords:** Climate-change impacts, Forest ecology, Tropical ecology

## Abstract

Increasing evidence shows that the functioning of the tropical forest biome is intimately related to the climate variability with some variables such as annual precipitation, temperature or seasonal water stress identified as key drivers of ecosystem dynamics. How tropical tree communities will respond to the future climate change is hard to predict primarily because several demographic processes act together to shape the forest ecosystem general behavior. To overcome this limitation, we used a joint individual-based model to simulate, over the next century, a tropical forest community experiencing the climate change expected in the Guiana Shield. The model is climate dependent: temperature, precipitation and water stress are used as predictors of the joint growth and mortality rates. We ran simulations for the next century using predictions of the IPCC 5AR, building three different climate scenarios (optimistic RCP2.6, intermediate, pessimistic RCP8.5) and a control (current climate). The basal area, above-ground fresh biomass, quadratic diameter, tree growth and mortality rates were then computed as summary statistics to characterize the resulting forest ecosystem. Whatever the scenario, all ecosystem process and structure variables exhibited decreasing values as compared to the control. A sensitivity analysis identified the temperature as the strongest climate driver of this behavior, highlighting a possible temperature-driven drop of 40% in average forest growth. This conclusion is alarming, as temperature rises have been consensually predicted by all climate scenarios of the IPCC 5AR. Our study highlights the potential slow-down danger that tropical forests will face in the Guiana Shield during the next century.

## Introduction

The tropical forests cover accounts for 25% of the terrestrial carbon pool, and therefore plays an essential role on carbon cycle and storage^1,2^. Higher atmospheric CO_2_ concentration might increase carbon uptake, maintaining the carbon sink historical role of tropical forests^3^. But recent droughts linked to *El Nino* phenomenon have weakened this carbon sink^4–7^, highlighting the dependence of tropical forest dynamics on the global Earth climate. On the other hand, tropical forest dynamic, through tree growth and mortality, itself impacts carbon storage and cycle, and provides important feedbacks on climate change. In this context, more and more efforts are being made to describe the long-term impact interplays between climate change and tropical forest functioning^8–13^. Recently, the impacts of exceptional droughts have been coaching more attention, first because droughts are predicted to be more frequent and severe in the tropics^14^, and second because tropical forests have already suffered from past severe droughts^15–17^. Massive tree mortality have been observed after droughts^18,19^, potentially caused by hydraulic failure and/or carbon starvation^20^, and affecting more severely large trees^19,21^. Beyond exceptional droughts and other long-term changes in water availability, temperatures are also expected to rise and the dry season length to increase over the next century in Amazonia^14,22^. These changes will likely impact tree dynamics^23,24^, and dynamic global vegetation models (DVGMs) sometimes predict a shift toward drier forests or even savannas^25^.

Coarse scale DGVMs allow efficient large-scale carbon cycle prediction with little input data, relying on a wide set of mechanistic assumptions^26^. These models were initially developed to simulate ecosystem carbon fluxes, they develop fast and are now used among other things to model nitrogen cycle^27^ or land management^28^, but also plant range shift^29^ or forest mortality^30^. However, DVGMs failed to predict observed regional patterns of tropical forest dynamics^31^ for two reasons. First, although DGVMs may model different major species or plant functional traits, they do not account for the huge tree diversity found in tropical forests so that they neglect the diverse functional strategies and the equally-diverse demographic strategies that shape tropical forest response to climate-induced disturbances^32–35^. Second, they are not demographic-explicit^30^. And we do know that it is essential to disentangle the ecosystem trajectory in a comprehensive process-based approach, i.e. by segregating the climate control on each demographic processes (growth, recruitment, mortality) as opposed to an all-in-one model in which only the ecosystem response is modeled, to reveal mechanisms underlying tropical forest response to disturbance and to make more robust predictions of the future trajectories^32,36,37^. To overcome these limitations, individual-based vegetation models provide a good framework to explore how climate and individual tree demographic strategy may interact and impact community tree dynamics. Managing diversity in these models can be done with functional traits that provide good proxies of the demographical strategies^38–41^ and at the same times reflects physiological differences in response to climate variations^42–45^.

In this paper, we investigate the potential impacts of climate change on long-term forest dynamics using an individual-based model calibrated with data from the Paracou long-term disturbance experiment, in the Guiana Shield. We simulated a tropical forest community under projected future climate scenarios. These simulations allow us to identify (1) the climate variables that will likely be responsible for most of the changes in forest dynamics, (2) the sensitive ecosystem processes and attributes that will be impacted, and (3) the way the forest structure will consequently change.

## Methods

### The SELVA individual-based model

The simulator SELVA is a an individual-based forest simulator set-up on the CAPSIS 4.0 Java platform^46,47^. In the simulator, individual growth, mortality and recruitment are described by sub-models on a two-year time step. Each tree *i* is described with the diameter at breast height (*DBH*_*i*_), the species (*s*_*i*_), a set of functional traits associated with each species (Table 1), and an individual vigor estimate. The simulator implements an already-parameterized joint growth-mortality model described earlier^48–50^, and a neutral recruitment model, based on the neutral assumption that each dead tree is replaced by a new recruited tree, respecting the proportion of each species in the metacommunity. The growth-mortality model used the individual tree parameters and climatic variables (Table 2) to compute individual growth and mortality probability at each time step. Details can be found in the Supplementary Information. The calibration of a precise recruitment model would necessitate more information about the small trees (diameter < 10 cm) and seedlings^51^, such information is often lacking in tropical forests. In the study site of Paracou, where no information is recorded for trees with DBH < 10 cm, a good modelling framework of recruitment is lacking. Therefore, we made the simplistic assumption of a neutral recruitment.

**Table 1:** The five functional traits used as proxies of ecological strategies in order to simulate a hyperdiverse tropical forest of the Guiana Shield under future climate scenarios.

Functional traits	Variable name	Range	Process
Maximum diameter (m)	*DBHmax*	[0.13; 1.11]	mortality and growth
Maximum height (dam)	*Hmax*	[0.8; 5.6]	mortality and growth
Trunk xylem density (g.cm^−3^)	*WD*	[0.28; 0.91]	mortality and growth
Laminar toughness (N)	*Tough*	[0.22; 11.4]	mortality
Foliar *δ*13*C* composition (%)	*δ*13*C*	[−3.61; −2.62]	growth

Description, name used in this study, range observed in our data set, and demographic process linked to the trait (growth or mortality, see Supplementary Information for details).

**Table 2:** The climate variables included in the growth-mortality model.

Variable	Abbreviation	*σ*	BASE	A		B		C	
			*μ*	*μ*	*δ*	*μ*	*δ*	*μ*	*δ*
Area over REW and <0.4	*A*_*under*_	8.1	20.2	20.2	0	22.9	0.0275	25.6	0.050
Precipitation (mm/2 years)	*Pre*	261.4	5858.6	5565.6	−2.99	5272.7	−5.98	4979.8	−8.97
Mean temperature (°C)	*Tmp*	0.26	26.5	27.8	0.013	29.4	0.029	31	0.046

Description, abbreviation used in this study, standard deviation (*σ*) observed in the actual (1991–2011) data set, actual mean value, predicted values for 2101 in the four scenarios (*μ*) and associated annual increment *δ*. Seasonal drought *A*_*under*_ was computed using a local water balance model^55^.

### Accounting for the individual vigor

The tree vigor was defined at the individual tree level and reflects the individual tree growth effect on the mortality model parameters, acknowledging that trees of a given species growing less than expected (as compared to individuals of the same species) have a far higher probability of dying and vice-versa^49^, the so-called dominance of the suppressed^52^. In our simulations, we used the individual vigor in two ways, reflecting two ways of seeing this intraspecific diversity in tree performance. First, we assume that the individual tree vigor is an endogenous property of a given tree and thus we sampled tree vigor once before starting the simulations. In this way, the individual tree vigor value will not be impacted by the climate-induced growth changes (model 1). Second, we assume that tree vigor is also under environmental control so that climate changes, by modifying the average growth of a given species, will also impact the individual vigor. In this way, we recalculated the individual tree vigor at each time step as the difference between the individual growth and the average species growth (model 2) and modified the mortality probability accordingly. Two versions of the model corresponding to these two hypotheses were used in this study. See Supplementary Information for mathematical details.

### Model inputs

To initialize the tree population, we used the tree inventories of the experimental site of Paracou, French Guiana, collected in 2001. The experimental site of Paracou (5°18′N,52°55′W) is a lowland tropical forest near Sinnamary, French Guiana. The forest is a typical Guiana shield forest, with dominant tree families including *Fabaceae*, *Chrysobalanaceae*, *Lecythidaceae*, and *Sapotaceae*. There are more than 700 woody species attaining 10 cm diameter at breast height (DBH) at the site. Six undisturbed plots of 6.25 hectares each totalizing 22,401 individual trees were used to constitute the initial population in the forest simulator. The functional traits used in this study are extracted from a large database collected in the Guiana Shield and described earlier^53,54^.

Three climate variables are needed to run the model^48^ (Table 2): a water stress estimator (*A*_*under*_), the total precipitation over two years (*Pre*) and the mean temperature (*tmp*). The water stress estimate *A*_*under*_ was based on a water balance model developed at our study site and taking the daily precipitation from the CRU as input data^55,56^. Four climate scenarios were investigated based on the scenarios of the IPCC report^14^. The first scenario (A) is equivalent to the RCP2.6, the second (B) is an intermediary scenario, and the third (C) is equivalent to the RCP8.5. The last scenario (BASE) is a control scenario that uses the current values of the climatic variables and assumes that they will remain stable over time.

At each time step, climate variables were sampled in a normal distribution where the mean changed over time, while the standard deviation remained the same, equal to the historical standard deviation. Historical values were computed between 1991 and 2011 using climatic data from the Climatic Research Unit (CRU) at the University of East Anglia^57^. The predicted mean temperatures (*Temp*) and rain (*Pre*) for the next century were computed using the prediction of the IPCC report^14^. The water stress estimator *A*_*under*_ was computed using an estimated change of the dry season length of plus two weeks over a century for the RCP8.5^22^ (Table 2). Details about the climatic scenarios can be found in the Supplementary Information.

### Model outputs

At each time step, we computed the community growth and mortality rates to track forest dynamics in time. To characterize the community structure at the end of the simulations, we computed the basal area per hectare (*BA*), the quadratic diameter (*QD*) and the above-ground fresh biomass (*AGFB*) with a local equation^58^.

### Sensitivity analysis

Different climate variables are used as drivers of the forest dynamics in our model, and these variables are predicted to evolve more or less drastically in the future. To disentangle which variables might be responsible for the forest dynamics evolution, we performed a variance based sensitivity analysis. This analysis consists in repetitions of simulation with varying intputs (climate variables) and study of the varying outputs (growth and mortality rates, *BA*, *QD*, and *AGFB*) with a sensitivity index computed with the variances of the outputs. The sensitivity analysis on the climate variables was conducted using a complete factorial design of 27 scenarios (3 scenarios, 3 climate variables). We ran the 27 scenarios 50 times and computed the first-order sensitivity index of Sobol (*S*_*i*_) for each climate variable *i*^59^:

$${S}_{i}=\frac{V[E({Y}_{j}|{X}_{i})]}{V({Y}_{j})},$$

where *X*_*i*_ is an input variable from the vector $X=({A}_{under},{Pre},TMP)$, and *Y*_*j*_ is an output variable from the vector $Y=(BA,morta,growth,AGFB,QD)$, $V[E({Y}_{j}|{X}_{i})]$ is the variance of the expected value (*E*) of the output variable *Y*_*j*_ knowing the input variable *X*_*i*_, and *V*(*Y*_*j*_) is the variance of the output variable *Y*_*j*_. The higher the sobol index, the higher the input variable impact on the output variable.

## Results

### Forest structure and dynamics

Average growth and mortality rates consistently decreased as the scenario became pessimistic (most pessimistic scenario is C), with the community mortality rate falling from 2 to 1.4% per 2 years and a community growth rate going from 0.25 to 0.16 mm per 2 years for the scenario C (Table 3). The forest structure variables *BA*, *QD*, and *AGBF* also decreased between scenario BASE and scenario C, but these reductions are less substantial than for the forest dynamic variables: *BA* mean is 30.7 in the scenario BASE and 30.1 in the scenario C, *QD* mean is 25.6 in the scenario BASE and 25.3 in the scenario C, and *AGBF* mean is 456 in the scenario BASE and 444 in the scenario C (Table 3).

**Table 3:** Summary statistics of the simulated model (versions 1 and 2), names used in the paper, definition, units and values.

Definition units		*growth*	*morta*	*BA*	*QD*	*AGFB*
		average growth rate mm.2 years^−1^	mortality rate %.2 years^−1^	basal area per hectare m^2^.ha^−1^	quadratic diameter cm	above ground fresh biomass t.ha^−1^
2001		0.26	2.1	30.4	25.1	436
1	BASE	0.25 ± 0.0018	2 ± 0.04	30.7 ± 0.33	25.6 ± 0.14	460 ± 6.4
	A	0.22 ± 0.0022	1.8 ± 0.03	30.6 ± 0.27	25.5 ± 0.11	450 ± 4.7
	B	0.19 ± 0.0019	1.6 ± 0.039	30.4 ± 0.25	25.5 ± 0.1	450 ± 4.7
	C	0.16 ± 0.0015	1.4 ± 0.028	30.1 ± 0.26	25.3 ± 0.11	440 ± 5.1
2	BASE	0.24 ± 0.0018	2 ± 0.04	27.5 ± 0.24	24.2 ± 0.1	395 ± 4.5
	A	0.22 ± 0.0019	1.9 ± 0.04	27.1 ± 0.24	24 ± 0.11	388 ± 4.6
	B	0.18 ± 0.0018	1.8 ± 0.04	26.5 ± 0.24	23.8 ± 0.11	378 ± 4.5
	C	0.16 ± 0.0015	1.7 ± 0.035	25.6 ± 0.25	23.5 ± 0.12	369 ± 4.7

Values are presented at the beginning of the simulation (2001) and mean values are presented at the end of the simulation (2101) for the four scenarios: BASE, A, B and C, for the versions 1 and 2 of the model and with standard deviations.

### On the role of individual tree vigor

**Figure 1:**
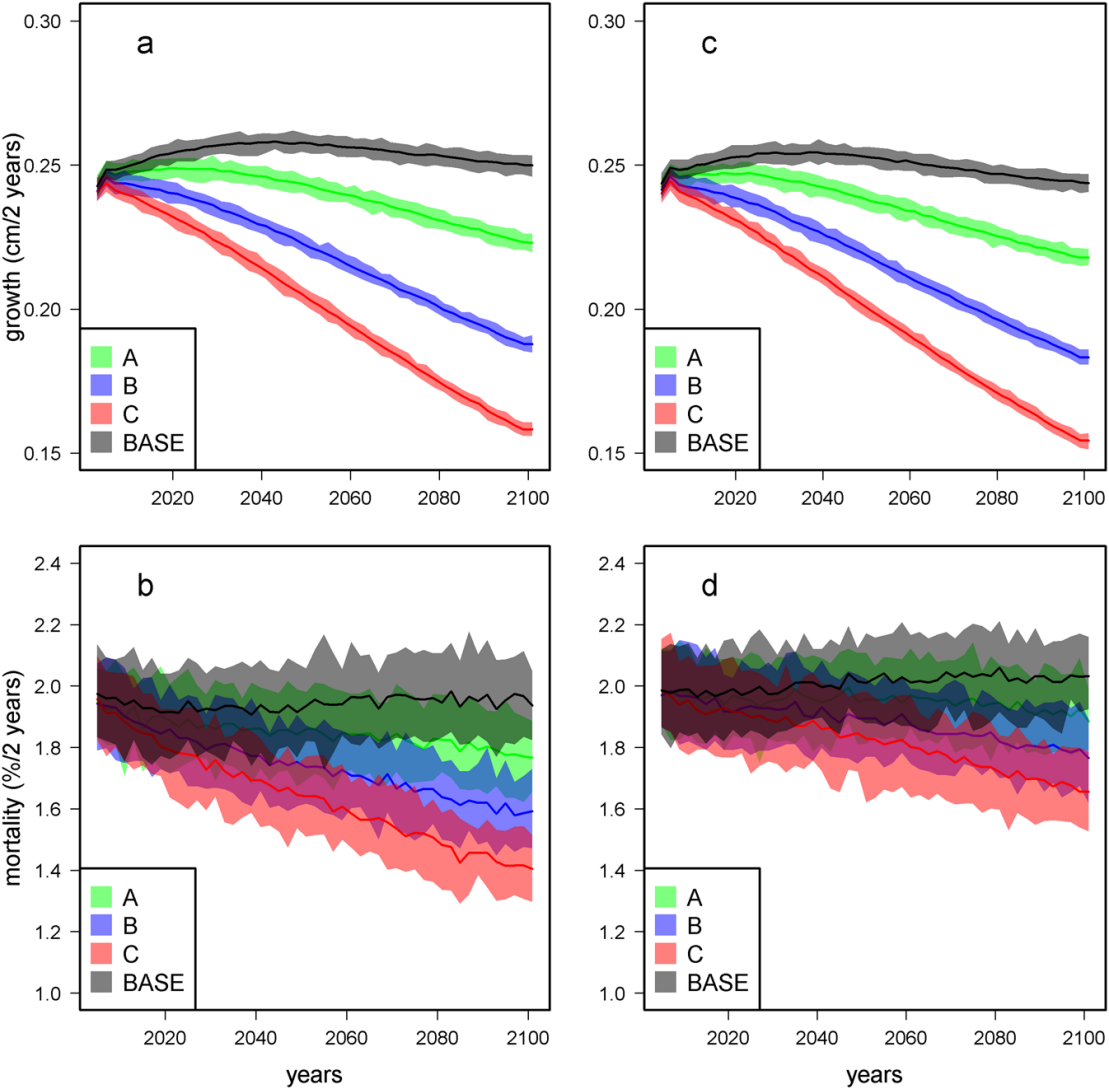
Evolution of the community-averaged growth and mortality rates for four climate scenarios and the two forest dynamic models. Growth rates (**a** and **c**) and mortality rates (**b** and **d**) for model 1 on the left (**a** and **b**) and model 2 on the right (**c** and **d**). Colored areas represent the 95% confidence interval. In model 1, we assumed that the vigor estimator is not impacted by climatic variables that impact the growth, whereas in model 2, we assumed that climatic variables that impact the community growth also impact the vigor and, consequently, the mortality. Scenario A is equivalent to the RCP2.6, B is an intermediary scenario, and C is equivalent to the RCP8.5. BASE is a control scenario that uses the current values of the climatic variables and assumes that they will remain stable over time (Table 2).

The two versions of the model correspond to two different individual tree vigor estimators (fixed at the beginning or updated during simulations). The reduction in growth is almost the same for models 1 and 2, and is quite progressive between 2001 and 2100 (Fig. 1). The reduction in mortality is much clearer for model 1 than for model 2, with a minimum for scenario C observed at 1.4% per 2 years for model 1 and a minimum of 1.7% per 2 years for model 2 (Fig. 1).

### Sensitivity analysis

**Figure 2:**
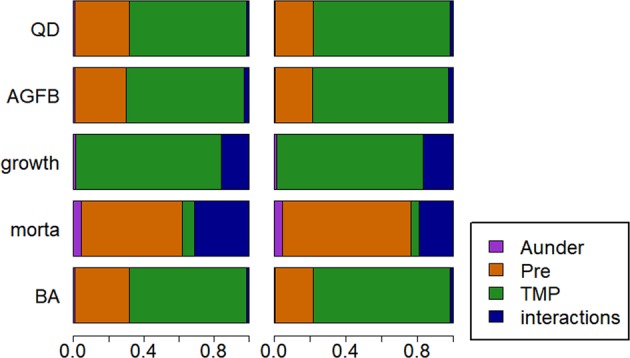
Results of the sensitivity analysis. Mean of the 50 Sobol indexes computed for each input and output variable. Inputs: *QD*: quadratic diameter, *AGBF*: above ground fresh biomass, *growth*: average growth rate, *morta*: mortality rate, *BA*: basal area. Outputs: *A*_*under*_: Area over REW and <0.4, *Pre*: precipitation, *TMP*: mean temperature, and interactions. Results of model 1 are on the left and model 2 on the right. Almost all outputs are primarily impacted by the temperature changes. Only mortality is strongly impacted by the precipitation changes.

Sensitivity analyses of models version 1 and 2 are very similar (Fig. 2). Growth was primarily controlled by changes in temperature, whereas mortality patterns were driven by precipitation. All the forest structure variables *BA*, *QD* and *AGFB* were mostly impacted by temperature (on average 67% of variance) and less by precipitation (between 29 and 31% of variance). Almost no effect of the drought estimator *A*_*under*_ was observed (0.7% of variance).

## Discussion

We used an individual-based forest model, where species diversity is approximated by functional traits and demographic processes are explicit, to simulate the future dynamics of the Paracou forest for the next century using predictions of the IPCC 5AR for three different climate scenarios (optimistic RCP2.6, intermediate, pessimistic RCP8.5) and a control (current climate). Whatever the scenario, all ecosystem processes and structure variables exhibited decreasing values as compared to the control, suggesting a general slow-down of the forest under climate change. A sensitivity analysis identified the temperature as the stronger climate driver of this behavior, highlighting a temperature-driven drop of 40% in average forest growth for the most pessimistic scenario (from 0.25 to 0.16 mm per .2 years^−1^, Table 3).

### Modeling limitations

As any forest simulators, the SELVA individual-based model is based on simplified assumptions. In our simulated communities, we took into account two major ecological processes, *i*.*e*. competition and response to stress, using the individual vigor. Indeed, the individual vigor can be seen as competitive vigor, the quality of how a tree is able to compete for resources, or it may also be used as capability to react to environmental stresses^49^. In model 2, the individual vigor is under environmental control so that climate changes, by modifying the average growth of a given species, will also impact the individual vigor and, then, forest dynamics. A major shortcoming of our approach is that, apart from the investigated climate drivers, other potentially important environmental variables were not explicitly modeled. Among others, the nutrient availability has often been highlighted as a major driver of forest dynamic in tropical forests^60^. In the Guiana Shield however, recent studies have concluded to a low control of soil nutrient availability on forest dynamics and suggested that nutrient-recycling mechanisms other than the direct absorption from soil (e.g. the nutrient uptake from litter, the resorption, or the storage of nutrients in the biomass), may be more important for forest functioning^61^. Hence we do recognize that SELVA present some limitations to study the future forest functioning but, because our modeling framework succeeded in reproducing the current forest structure and dynamics from real data (see Supplementary Information), we are quite confident in the model ability to explore their future evolution.

### On the importance of tree vigor

The two investigated models differed in the ways the tree individual vigor was implemented. In model 1, the reduction of growth due to higher temperature in time did not influence mortality rates so that the decreasing mortality rates was only due to rain diminution. In model 2, the reduction of growth due to higher temperature induced a reduction of the tree vigor which increased mortality rates. This compensates the effect of rain diminution itself and, all in all, leads to a less marked decrease in mortality rates than in model 1. This result highlights the key role of the individual tree vigor^49^, a component still insufficiently taken into consideration in forest models^52^. Model 1 looks better adapted to simulate the actual dynamics observed in our study site in French Guiana, as no evident correlation has been empirically found between temperature and mortality rates in our studied forests^48^. This means that the rise in temperature would solely impacts the growth. However, strong links between growth slow-down and mortality risks are already well documented^62^, and past growth, a surrogate of our tree vigor, is sometimes used as a predictor of mortality in forest models^63^. During an experimental throughfall exclusion in Brazil, a decrease in growth was observed^64^, and followed a few years later by an increase in mortality rates^65^. These experimental results are more consistent with model 2, *i*.*e*. where a decrease in tree vigor translates, at next time step, into an increase in mortality risk. This makes the choice between model 1 and 2 difficult and we have to admit that we almost ignore how this tree vigor component will behave in the next century under the climate pressures that will be different from those currently observed. The reality will probably fall between models 1 and 2, and therefore these two models are useful to explore the possible futures and to measure the impacts of the different hypotheses we put forward to construct our simulations.

### Temperature is the main driver of future forest dynamic

Temperature rise is by far the strongest driver of almost all summary statistics while precipitation variability primarily influences mortality rates only. First and foremost, our results must be considered with caution because the simulated ranges of climatic variables solely depends on the IPCC 5AR predictions. According to the latter, the relative changes in temperature values will be higher than the relative changes for precipitation and water stress, and this clearly underlies our results (Fig. 2). Nevertheless, our results highlight the important role of future temperature rises in tropical forest dynamic and structure, confirming previous studies^66,67^. In our simulations, growth is the most impacted demographic process, and this slowing-down dynamics implies, all else being equal, a substantial reduction in above-ground biomass, quadratic diameter and basal area. If, as highlighted by the results from model 2, the temperature-driven growth reduction leads to higher mortality rates, the forest community structure will significantly change with few large old canopy trees and more small slow-growing trees, with possible consequences for *e*.*g*. ecosystem water uptake from deep soil layers during dry season^68^. This community change will impact the basal area (from 30.1 m^2^.ha^−1^ for scenario C with model 1 to 26 m^2^.ha^−1^ for scenario C with model 2) and the above-ground fresh biomass (from 444 t.ha^−1^ for scenario C with model 1 to 369 t.ha^−1^ for scenario C with model 2). In order to be concrete, temperature is expected to rise of 4.5 °C during the next century in the Guiana Shield. Such temperature can drastically affect photosynthesis by causing irreversible damage to the functioning of leaves^4^ and we have to admit we are in uncharted ground because, currently, no forests in the world exist in areas with mean temperatures of 31 °C. Nevertheless, we do know, from a leaf physiologist perspective, that as the temperature rises, the velocity of reacting molecules increases, leading to more rapid reaction rates but also to damage of the tertiary structures of the enzymes^69^. These two processes lead to the well-known bell-shaped curve of growth response to temperature^70^. Temperature also affect photosynthesis in a more indirect manner, through leaf temperatures defining the magnitude of the leaf-to-air vapor pressure difference, a key factor influencing stomatal conductances^69^. In the tropical environment of the Guiana Shield, as temperatures are already very high, rising temperatures will imply lower growth.

### Uncertain impacts of precipitation changes

The predicted reduction of precipitation spearheads a noticeable reduction in mortality rates. This counter-intuitive results is however supported by a growing common understanding that strong winds and heavy rainfalls associated with severe convective storms are the dominant natural drivers of tree mortality in the Amazon^71,72^. This precipitation-driven mortality is obvious at Paracou where the proportion of fallen trees, relatively to standing death, is higher during the most rainy years^48^, trees being more vulnerable to uprooting when soil is water-saturated^73^. Consequently, the predicted decrease of precipitation implies a decrease in mortality rates in the simulated forest communities. But the IPCC AR5 also forecasts an intensification of abundant rain events in the tropics^14^, that may play the inverse role, increasing mortality rates. The problem is that such punctual and rare events are currently not well quantified, and relations between mortality and extreme events are statistically complex to model^20,74^. This makes mortality a crucial demographic process upon which we need to focus our research effort.

## Conclusion

Our study highlights the potential slow-down danger that tropical forests will face in the Guiana Shield during the next century and this conclusion is alarming, as temperature rises have been consensually predicted by all climate scenarios of the IPCC 5AR.

## Supplementary information

Supplementary Materials
