# Supplementary material for: Temperature rising would slow down tropical forest dynamic in the Guiana Shield
Source: Sci Rep. 2019 Jul 15;9:10235. doi: 10.1038/s41598-019-46597-8 (PMC6629855; doi:10.1038/s41598-019-46597-8)
Supplement: Supplementary file 1 — Supplementary Materials [file 41598_2019_46597_MOESM1_ESM.pdf]

Supplementary information

Temperature rising would slow down forest dynamic in the Guiana Shield

Mélaine Aubry-Kientz, Vivien Rossi, Guillaume Cornu, Fabien Wagner, Bruno Hérault

Model equations

The model of growth and mortality used in the simulator is based on [1] and is described by the following equations.

$$\begin{aligned} p_{i,s,t} =& logit^{-1}\Bigg(\theta_1 \times A_{under,t} + \theta_2 \times Pre_t + \theta_3 \times Vigour_{i,s,t} \\ &+ \theta_4 \times \frac{DBH_{i,s,t-1}}{DBHmax_s} + \theta_5 \times \left(\frac{DBH_{i,s,t-1}}{DBHmax_s}\right)^2 \\ &+ \theta_6 \times Hmax_s + \theta_7 \times WD_s + \theta_8 \times Tough_s\Bigg) \qquad (1) \end{aligned}$$

and

$$\begin{aligned} log(\widehat{AGR}_{i,s,t-1} + 1) =& (\theta_9 \times tmp_{t-1} + \theta_{10} \times A_{under,t-1} \\ &+ \theta_{11} \times A_{under,t-1} \times (WD_{max} - WD_s) + \theta_{12} \times A_{under,t-1} \times DBH_{i,t-2} \\ &+ \theta_{13} \times DBHmax_s + \theta_{14} \times WD_s + \theta_{15} \times Hmax_s + \theta_{16} \times \delta 13C_s) \\ &\times exp\left(-\frac{1}{2}\left(\frac{log\left(\frac{DBH_{i,t-2}}{\theta_{17} \times DBHmax_s}\right)}{\theta_{18} \times WD_s}\right)^2\right), \qquad (2) \end{aligned}$$

and

$$\begin{aligned} log(AGR_{i,s,t-1} + 1) =& log(\widehat{AGR}_{i,s,t-1} + 1) + \varepsilon_i \qquad (3) \\ & \text{with } \varepsilon_i = \alpha_i + \beta, \\ & \text{and } \alpha_i \sim \mathcal{N}(0, \theta_{19}), \text{ and } \beta \sim \mathcal{N}(0, \theta_{20}), \end{aligned}$$

where $p_{i,s,t}$ is the probability of the death of tree i of species s between time $t-1$ and t; $Vigour_{i,s,t}$ is the vigour estimator for tree i of species s between time $t-1$ and t; and $\widehat{AGR}_{i,s,t-1}$ is the predicted growth between time $t-2$ and time $t-1$. $AGR_{i,s,t-1}$ is the observed growth between time $t-2$ and time $t-1$; $DBHmax_s$, $Hmax_s$, $Ortho_s$, WD_s, $Tough_s$ and $\delta 13C_s$ are functional traits of species s to which tree i belongs. $\theta_1, \theta_2, \cdots \theta_{18}$ are parameters sampled in normal distributions with the mean and standard deviation computed using the MCMC method developed in [1] (Tables 1 and 2). $A_{under,t}$, tmp_t and

Pre_t are the climatic variables computed between times $t-2$ and $t-1$ [1]. $A_{under,t-1} \times (WD_{max} - WD_s)$ is an interaction term to account for the sensitivity of a tree with low wood density to water stress; and $A_{under,t-1} \times DBH_{i,t-2}$ is an interaction term to account for the sensitivity of a big tree to water stress [1]. α_i is an individual effect sampled in a normal law centered on 0, and β is a residual error sampled in a normal law centered on 0.

Vigour estimator

In the model version 1, the vigour estimator is computed once per individual tree as the error of the growth model using the parameterized Equation (4).

$$Vigour_{i,s,t} = log\left(\frac{AGR+1}{\widehat{AGR}+1}\right) = log(AGR+1) - log(\widehat{AGR}+1) \qquad (4)$$

In the model version 2, an alternative definition of the vigour is used. We assumed that tree vigor is also under environmental control so that climate changes, by modifying the average growth of a given species, will also impact the individual vigor. In this way, we recalculated the individual tree vigor at each time step as the difference between the individual growth and the average species growth using Equation (5).

$$AltVig_{i,s,t} = log(AGR+1) - \Big(log(\widehat{AGR}+1) - (\theta_9 \times Pre_{t-1} + \theta_{10} \times A_{under,t-1} + \theta_{11} \times A_{under,t-1} \times (WD_{max} - WD_s) + \theta_{12} \times A_{under,t-1} \times DBH_{i,t-1})\Big) \qquad (5)$$

In Equation (5), if a tree's growth is negatively impacted by climate drivers, this will impact the vigour in the same way. This hypothesis implies that a climate predictor that reduces growth automatically increases mortality.

The vigour estimator is of particular interest for simulations. Indeed, this vigour may be described as an individual effect of genetics or a microsite effect, for instance, but it may also be described as a temporal effect of more or less favorable years. On the other hand, vigour may be described as an additional error, independently sampled at each time step, and the result of multiple environmental causes. To take these two effects into account, the computed term of error ε_i was modelled by a mixed model with an individual effect, a time effect and a residual error. The total variance of the vigour estimator was mostly due to the individual effect (42%), whereas no time effect was observed (0.005% of variance). The residual error accounted for more than one half of the variance (57%). To apply these results in the simulator, when a tree was recruited, an individual error was sampled and did not change during the life of the tree (α_i). The additional error (β) was sampled at each time step and added to the individual error to compute the vigour estimator.

Climate scenarios

The climate variables for each year are sampled from a normal distribution. The mean changes at each time step, with a linear function as described in the following equation.

$$clim_{\Delta t,s} \sim \mathcal{N}(clim_0 + \Delta t \times \delta_{clim,s}, \sigma^2_{clim}) \quad (6)$$

where $clim_{\Delta t,s}$ is the value of the climatic variable at time 2001 + Δt for the scenario s; $clim_0$ is the reference mean value for climatic variable $clim$; $\delta_{clim,s}$ is the increment of climatic variable $clim$ for the scenario s computed hypothesizing a linear increase with time to reach the predicted values in 2101; and σ^2_{clim} is the variance of climatic variable $clim$.

For instance, to compute the water stress estimator A_{under} in year 2100 and for scenario C, we used: $A_{under,2100,C} \sim \mathcal{N}(A_{under,0} + 100 \times \delta_{A_{under},C}, \sigma^2_{A_{under}})$

The scenarios were created using values of temperature ($Temp$) and precipitation (Pre) found in the IPCC fifth assessment report [3]. This report forecasts an increase in temperature between 1 and 1.5 °C for the scenario RCP2.6 and an increase in temperature between 4 and 4 °C for the scenario RCP8.5 for the next century. Forecasts of precipitation are between 0 and -10% for the scenario RCP2.6 and between -10 and -20% for the scenario RCP8.5. To compute the water stress estimator A_{under}, a linear regression was used to link A_{under} with the length of the dry season in days. Estimated change of the dry season length is plus two weeks for the RCP8.5 [2], while no consensual change was observed for the RCP2.6 that corresponds to our scenario A. This 2-week increase is equivalent to an increase of 5.6 units of the water stress estimator A_{under} for the scenario C, and no change for the scenario A.

Parameters for the simulator

Table 1: Parameters used in the simulator, version 1. Each parameter is sampled at each time step in a normal distribution with mean and standard deviation computed in [1]. θ_{19} and θ_{20} are the standard deviation used to compute the individual effect and the residual error of the growth model.

parameter	mean	standard deviation
θ_1	- 0.0062	0.002
θ_2	0.00032	0.000074
θ_3	- 0.53	0.024
θ_4	- 0.6	0.21
θ_5	0.48	0.13
θ_6	- 0.4	0.022
θ_7	- 2.8	0.15
θ_8	- 0.36	0.031
θ_9	- 0.076	0.011
θ_{10}	- 0.0056	0.002
θ_{11}	0.0066	0.0027
θ_{12}	- 0.00016	0.000021
θ_{13}	1.8	0.018
θ_{14}	- 0.37	0.033
θ_{15}	- 0.062	0.0039
θ_{16}	- 0.2	0.0078
θ_{17}	0.79	0.022
θ_{18}	2.35	0.052
θ_{19}	0.58	-
θ_{20}	0.5	-

Table 2: Parameters used in the simulator, version 2. Each parameter is sampled at each time step in a normal distribution with mean and standard deviation computed in [1]. θ_{19} and θ_{20} are the standard deviation used to compute the individual effect and the residual error of the growth model.

parameter	mean	standard deviation
θ_1	- 0.0066	0.0021
θ_2	0.0003	0.000072
θ_3	- 0.52	0.024
θ_4	- 0.6	0.22
θ_5	0.48	0.14
θ_6	- 0.4	0.022
θ_7	- 2.8	0.16
θ_8	- 0.36	0.031
θ_9	- 0.076	0.012
θ_{10}	- 0.0054	0.0018
θ_{11}	0.0064	0.0025
θ_{12}	- 0.00017	0.000021
θ_{13}	1.8	0.019
θ_{14}	- 0.37	0.031
θ_{15}	- 0.062	0.0043
θ_{16}	- 0.2	0.0068
θ_{17}	0.79	0.023
θ_{18}	2.35	0.053
θ_{19}	0.58	-
θ_{20}	0.5	-

Model validation

The control scenario BASE has been used to reproduce the forest dynamics of Paracou between 1993 and 2009. Ten simulations have been realized and different outputs computed: basal area, quadratic diameter, diameter median and 90% quantile, diameter growth, and mortality rate

Table 3: Results of the simulations for scenario BASE between 1993 and 2009: median and 10-90 percentiles computed for the ten simulations (Sim), and for the field observations (Obs).

	Basal area ($m^2.ha^{-1}$)	Quadratic diameter (cm)	Median diameter (cm)	Diameter 90% (cm)	Growth (cm)	Mortality rate
Sim median	29.90	25.64	17.75	39.35	0.14	0.018
Sim 10%	28.99	25.26	17.51	38.77	0.09	0.015
Sim 90%	30.93	26.07	18.11	40.02	0.20	0.022
Obs median	31.16	25.02	17.35	38.83	0.16	0.015
Obs 10%	30.80	24.94	17.19	38.48	0.00	0.011
Obs 90%	31.48	25.29	17.51	39.22	0.64	0.019

References

[1] M. Aubry-Kientz, V. Rossi, F. Wagner, and B. Hérault. Identifying climatic drivers of tropical forest dynamics. *Biogeosciences*, 12(19):5583–5596, 2015.

[2] E. Joetzjer, H. Douville, C. Delire, and P. Ciais. Present-day and future Amazonian precipitation in global climate models: CMIP5 versus CMIP3. *Climate Dynamics*, 41(11-12):2921–2936, 2013.

[3] T. F. Stocker, D. Qin, G.-K. Plattner, M. Tignor, S. K. Allen, J. Boschung, A. Nauels, Y. Xia, V. Bex, and P. M. Midgley (eds.). IPCC, 2013: Climate Change 2013: The Physical Science Basis. Contributi on of Working Group I to the Fifth Assessment Report of the Intergovernmental Panel on Climate Change. *Cambridge University Press, Cambridge, United Kingdom and New York, NY, USA*, 2013.
